# Effect of complementary food supplementation on breastfeeding and home diet in rural Bangladeshi children^1^,^2^

**DOI:** 10.3945/ajcn.116.135509

**Published:** 2016-09-28

**Authors:** Rebecca K Campbell, Kristen M Hurley, Abu Ahmed Shamim, Saijuddin Shaikh, Zaynah T Chowdhury, Sucheta Mehra, Saskia de Pee, Tahmeed Ahmed, Keith P West, Parul Christian

**Affiliations:** 3 Department of International Health, Johns Hopkins Bloomberg School of Public Health, Baltimore, MD;; 4 FHI360, Dhaka, Bangladesh;; 5 Nutrition Division, United Nations World Food Program, Rome, Italy;; 6 Center for Nutrition and Food Security, International Centre for Diarrhoeal Disease Research, Bangladesh, Dhaka, Bangladesh

**Keywords:** Bangladesh, breastfeeding, complementary food, dietary diversity, growth, stunting, supplementation

## Abstract

**Background:** Complementary food supplements (CFSs) can enhance growth where stunting is common, but substitution for the usual diet may reduce observed benefits.

**Objective:** We aimed to characterize dietary diversity from home foods in a CFS efficacy trial and determine whether supplementation reduced breastfeeding frequency or displaced home foods.

**Design:** In a cluster-randomized controlled trial in rural Bangladesh, children (*n* = 5499) received, for 1 y starting at age 6 mo, periodic child feeding counseling for mothers (control) or counseling plus 1 of 4 CFSs fed as a daily snack. Breastfeeding status and past 24-h diet were assessed at enrollment and every 3 mo thereafter until 18 mo of age. A 7–food group dietary diversity score (DDS) was calculated from home foods only, and a DDS ≥4 constituted minimum dietary diversity (MDD).

**Results:** Most children (97%) were breastfed through 18 mo of age, and 24-h breastfeeding frequency did not differ by supplementation group. Child dietary diversity was low; only 51% of children met the MDD by 18 mo. Rice, potatoes, and biscuits (cookies) were the most frequently consumed foods, whereas the legumes, dairy, eggs, and vitamin A–rich fruit and vegetable food groups were each consumed by <50% of children. The odds of meeting the MDD through the consumption of home foods were equal or greater in the supplemented groups compared with the control group at all ages. High socioeconomic status and any maternal education were associated with increased odds of MDD at age 18 mo, whereas child sex and household food security were not associated with MDD.

**Conclusions:** In a setting where daily complementary food supplementation improved linear growth, there was no evidence that supplementation displaced breastfeeding or home foods, and the supplementation may have improved dietary diversity. Pathways by which supplementation with fortified foods may enhance dietary diversity, such as an improved appetite and increased body size, need elucidation. This trial was registered at clinicaltrials.gov as NCT01562379.

## INTRODUCTION

In low- and middle-income countries where prevalent stunting persists and contributes to high rates of morbidity and mortality in childhood and throughout the life course (1–3), diet quality and feeding practices contribute to children’s risk of stunting (4). The dietary diversity score (DDS)^7^ is widely used as an indicator of diet quality because it is easy to measure and is consistently associated with nutrient intakes and nutritional status in adults and young children (5, 6). Feeding practices such as continued breastfeeding, proper timing of complementary food introduction, and meal frequency may also be associated with better nutritional status during the complementary feeding period, although some mixed findings have been reported (4, 7).

Complementary food supplements (CFSs) are deployed with the aim of remedying an inadequate diet quality to support healthy growth. Large-quantity, lipid-based, ready-to-use therapeutic foods were first developed for the home treatment of acute malnutrition, but more recently, their formulations have been adjusted to create small- and medium-quantity, lipid-based nutrient supplements to prevent undernutrition in children at high risk and to treat moderate acute malnutrition (8, 9). Concerns that CFSs may be substituted for breastmilk or for other complementary home foods have contributed to reservations about distributing CFSs programmatically. To our knowledge, no direct experimental evidence of that substitution exists, but evidence of a tradeoff between complementary food energy density and breastmilk intake (10, 11) has suggested that increasing the energy density of the diet with CFSs may lead to reduced breastmilk consumption. Two studies in Malawi also reported reduced intake of staple foods in children who received fortified blended food supplements, although children who were given lipid-based supplements had no decline in their intake of other complementary foods (12, 13). In other trials of lipid-based nutrient supplements, substitution for foods or breastmilk was not observed (14–16).

In rural northwest Bangladesh, using a cluster-randomized controlled trial, we tested 4 formulations of CFSs for preventing stunting. Three of the supplements, including 2 supplements that were developed and produced in-country, were shown to slow the deceleration of linear growth from 6 to 18 mo of age compared with the unsupplemented control group, whereas the effect of the fortified wheat-soy blend (WSB++) was not significantly different from that of the control (17). Because the CFSs were deployed on the premise that inadequate dietary intakes were constraining growth during the vulnerable complementary feeding period, the aims of the current study were to *1*) characterize the dietary diversity of home diets of children in control and supplemented groups, *2*) assess the effect of providing daily small-to-medium quantity CFSs on the dietary diversity and breastfeeding frequency of participating children, and *3*) examine factors associated with adequate dietary diversity in children at 18 mo of age.

## METHODS

### Setting

The trial (clinicaltrials.gov; NCT01562379) was conducted from 2012 until 2014 in the Gaibandha and Rangpur districts of rural northwest Bangladesh. The study area was selected to be broadly representative of health and nutrition conditions throughout the Gangetic flood plains of South Asia and has been host to previous randomized controlled supplementation trials and numerous other observational studies of maternal and child health (18–20). The details of the trial have been published elsewhere (17). Briefly, 596 geographic clusters (called sectors) were randomly assigned to 1 of 5 supplement arms. The 5 groups were as follows: a group who received child feeding counseling (CFC) only (control) and groups who received CFC plus 1 of 4 CFSs. Two of the CFS products were developed and produced in-country, one of which was made of chickpeas (chickpea CFS) and the other of rice and lentils (rice and lentil CFS), and both products were provided as ready-to-eat pastes in single-serving sachets. The third product was WSB++ that was supplied by the World Food Programme and was fed to children as a porridge, and the fourth was a commercially produced, ready-to-eat, peanut-based product (Plumpy’doz; Nutriset). The supplements were isocaloric and approximately balanced in their micronutrient contents. Children aged 12–18 mo received 250-kcal portions of a supplement per day, while children aged 6–11 mo were given 125-kcal portions per day according to guidelines for the use of Plumpy’doz to prevent undernutrition. Supplementation took place over 1 y from enrollment at age 6 mo until the child reached his or her 18-mo birthday. All children who were living within the designated study area and who reached age 6 mo during the enrollment period were eligible to enroll.

CFC was delivered to mothers of all participating children (regardless of the study arm) by trained counselors on 9 occasions throughout the 1-y supplementation period (monthly from ages 6–10 mo and at months 12, 14, and 16). Counseling messages were based on modules that were developed for the Alive and Thrive project in Bangladesh and included messaging about continued breastfeeding, appropriate types, diversity, and quantity of complementary foods, hand washing and food-preparation hygiene, and continued feeding during illness. A female community worker visited participating households 2 times/wk to record daily supplement adherence through maternal report of daily quantities fed and observation of remaining supplement portions, and replenished CFSs 1 time/wk.

The primary outcomes for the supplementation trial were gains in the length-for-age *z* score (LAZ) and reduced rates of stunting at age 18 mo in the supplemented groups compared with in the control group and noninferiority in terms of the LAZ and stunting of the chickpea, rice and lentil, and WSB++ groups compared with that of the Plumpy'doz group. The necessary sample size was determined according to these aims, and the target enrollment in the CFC-only and Plumpy'doz arms were inflated by a factor of 1.7 to allow for multiple comparisons between groups. Sample size calculations assumed an α = 0.05, β = 0.10, and a 5% loss to follow-up and were adjusted for the cluster-randomized design with a design effect of 1.5. The expected mean ± SD LAZ at endline was based on published values in the literature.

Enrollment interviews were conducted on or soon after the participating child’s 6-mo birthday by a trained data-collection team. The interview consisted of detailed child characteristics including anthropometric measures, morbidity, and vaccination history. Household composition, socioeconomic status (SES), and parental characteristics were also assessed in the enrollment interview. The SES assessment included questions about the house structure and size, the family’s land, livestock, and durable-asset ownership, parental education and employment details, and perceptions of household food security based on the 9-item Food Access Survey Tool method (21).

Child diet was assessed with the use of a semistructured 24-h recall that was administered to all participants at the 6-, 9-, 12-, 15-, and 18-mo interviews. The questionnaire contained 29 food items that are commonly consumed by children in this setting and age group. For each food item, the interviewer asked the mother or primary caregiver, “From yesterday morning to today morning, has the child been fed [the food item]?” If the answer was yes, the interviewer asked how much of the item was fed to the child and recorded the quantity and unit of measurement using standard measures. After completing the listed foods, the interviewer asked if the child had eaten any additional foods (not contained in the food list) in the past 24 h. The form allowed the interviewer to record ≤6 additional foods and the quantities consumed. The food list was developed based on frequently consumed foods in this age group that were identified from dietary data that were collected in a recent study in this population (20). In the supplemented groups, mothers were instructed not to report the CFS in the diet recall.

Breastfeeding status was assessed at each interview with the following 2 questions: “Are you currently breastfeeding this child?” (recorded as yes or no) and “During all of yesterday and last night, how many times did you breastfeed this child?” (recorded as 1–10, 11–20, or ≥21 times) as was done previously in this setting.

### Data management and index development

The DDS was used as an indicator of diet quality on the basis of previous studies in Bangladesh that have linked the DDS to dietary nutrient intakes (22) and child nutritional status (23). A 7-item DDS was calculated for each child and interview by summing the number of food groups that the child consumed according to WHO/UNICEF guidelines (24) such that the DDS ranged from 0 to 7. The food groups were *1*) grains and starchy roots, *2*) legumes and nuts, *3*) dairy, *4*) meat and fish, *5*) eggs, *6*) vitamin A–rich fruit and vegetables, and *7*) other fruit and vegetables. All questionnaire-based and specified other foods were assigned to food groups. Mixed dishes were counted toward all groups represented by their ingredients [i.e., a child consuming suji (porridge with milk) was considered to have eaten grains and dairy]. A minimum dietary diversity (MDD) was defined as a DDS ≥4 according to WHO guidelines (24). CFSs were not assigned to any food group for this analysis nor were they included in the calculations of the DDS or MDD because the objective of this analysis was to determine whether the DDS and MDD from home foods differed by supplement group. Breastfeeding frequency was coded as none (not currently breastfeeding) or 1–10, 11–20, or ≥21 times.

SES characteristics were defined for this analysis as follows. The mother’s reported years of education completed were collapsed to create 4 levels as follows: none, 1–9 y, secondary school certificate, and ≥11 y. The 9-item household food-security questionnaire was collapsed into a household food insecurity index (HFI) with possible scores ranging from 9 to 36 and with higher values indicative of more severe food insecurity. The HFI was further categorized as no (HFI = 9), mild (HFI >9 to <16), and severe (HFI ≥16) food insecurity as was done previously for this data on the basis of an examination of value distributions (17). The living standards index (LSI), which is a composite SES score of household assets and dwelling-structure characteristics that was developed for this study site (25), was calculated and dichotomized around the internal median.

The study was approved by the Institutional Review Boards of the Johns Hopkins Bloomberg School of Public Health and the International Center for Diarrhoeal Disease Research, Bangladesh. Written consent was obtained from the mother or caregiver of each child before enrollment.

### Statistical analysis

Baseline differences in breastfeeding, diet, and other child and household characteristics at enrollment (6 mo of age) were examined by supplementation arm. Group-wise differences were quantified with the use of linear regression models with indicator variables for the supplementation groups and robust SEs to account for design effects. Differences in breastfeeding frequency between supplementation groups were tested with ordinal logistic regressions with the breastfeeding-frequency categories (none or 1–10, 11–20, or ≥21 times) as dependent variables, indicator variables for the supplementation groups as independent variables, and robust SEs for clustering by sector.

To assess the supplementation effects on the odds of reaching the MDD from home foods, mixed-effects logistic regression models were developed with fixed effects for the supplementation group and time point (child age) modeled with indicator variables and supplementation group-by-age interaction terms. Random intercepts were allowed for each child and sector.

To examine child and household factors that were associated with meeting the MDD at 18 mo of age, we used logistic regression models with SEs that were adjusted for clustering by sector. Multivariable logistic models were fit with MDD as the outcome and all of the child and household factors in one model.

Participants with missing data were excluded from the relevant analyses; no missing values were imputed. All analyses were conducted with Stata software (version 14.1; StataCorp LP).

## RESULTS

**FIGURE 1:**
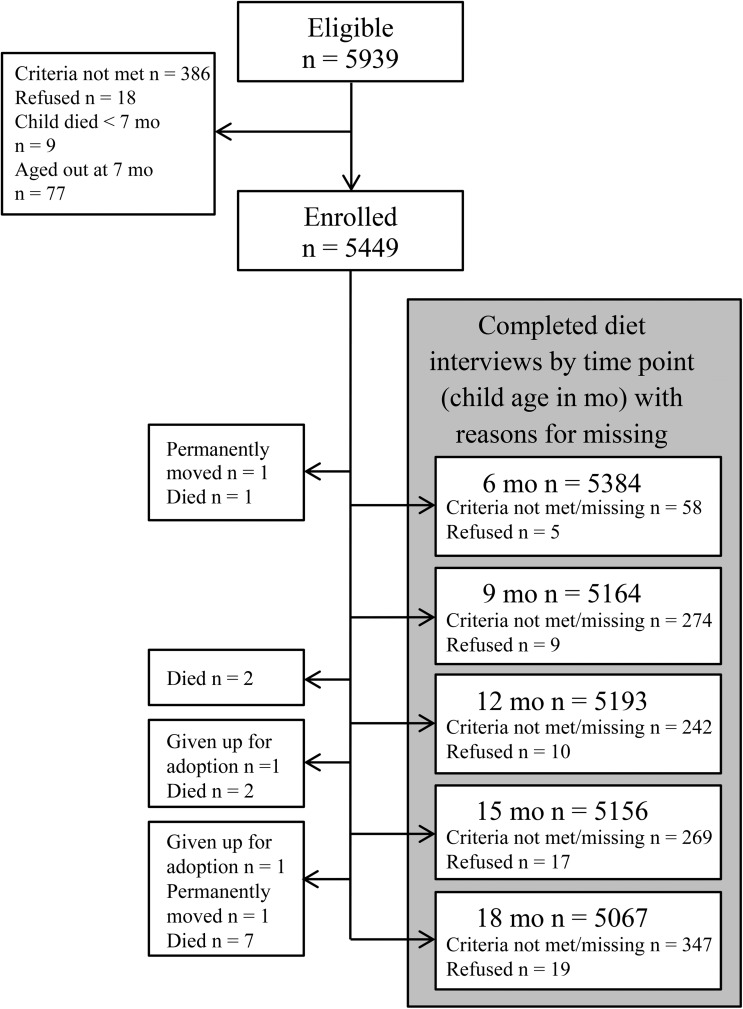
Flow diagram for available dietary data of children enrolled in a randomized controlled trial of complementary food supplements in rural Bangladesh. Reasons for missing that are included in individual interview boxes pertain to that interview only, whereas those in boxes at the left of the figure apply to all subsequent interviews.

The study enrolled 5449 children, 92% of those eligible on the basis of previous household surveys for the enumeration of study-area births and young children. Of children enrolled, 93–99% had complete diet interviews at ages 6, 9, 12, 15, and 18 mo, and 4710 children (86%) had complete interviews for all time points (**Figure 1**). At enrollment (age 6 mo), participants were comparable by supplement group on a number of individual and household factors (**Table 1**) (17). Across groups, ∼50% of children were girls, the mean age was 6.4 mo, and 25.5% were stunted. Breastfeeding was nearly universal at enrollment (99.1%). The DDS was low with a median DDS of 1 (IQR: 0–2), and 5.2% of children met the MDD cutoff (≥4 food groups consumed).

**TABLE 1:** Baseline child and household characteristics of participants in a randomized trial of complementary food supplements by assigned study arm^1^

Characteristic	CFC only	Chickpea	Plumpy'doz	Rice and lentil	WSB++
*n*	1436	839	1490	823	849
Child characteristic					
Age,^2^ mo	6.13 ± 0.00^3^	6.24 ± 0.25	6.26 ± 0.26	6.25 ± 0.25	6.26 ± 0.27
LAZ^4^	−1.33 ± 1.04	−1.33 ± 1.08	−1.34 ± 1.05	−1.4 ± 1.09	−1.43 ± 1.10
WLZ	−0.40 ± 1.03	−0.42 ± 1.08	−0.36 ± 1.08	−0.38 ± 1.00	−0.44 ± 1.00
WAZ	−1.19 ± 1.08	−1.21 ± 1.10	−1.17 ± 1.09	−1.22 ± 0.97	−1.28 ± 1.07
Sex, M, *n* (%)	686 (48.6)	419 (50.5)	767 (51.8)	400 (49.0)	419 (49.5)
Parental and household characteristic					
Household size	5.0 ± 1.7	5.1 ± 1.9	5.0 ± 1.8	5.0 ± 1.8	5.0 ± 1.8
Maternal education, *n* (%)					
None	357 (25.1)	193 (23.1)	361 (24.3)	190 (23.3)	197 (23.6)
1–9 y	916 (64.3)	543 (64.9)	950 (64)	546 (66.9)	533 (63.8)
SSC passed	77 (5.4)	47 (5.6)	74 (5)	31 (3.8)	41 (4.9)
≥11 y	75 (5.3)	54 (6.4)	99 (6.7)	49 (6)	65 (7.8)
HFI, *n* (%)					
None	712 (49.9)	432 (51.6)	760 (51.2)	429 (52.5)	418 (49.8)
Mild	520 (36.4)	294 (35.1)	537 (36.2)	291 (35.6)	309 (36.8)
Severe	196 (13.7)	111 (13.3)	187 (12.6)	97 (11.9)	112 (13.4)
Assets, *n* (%)					
Cattle^5^	701 (49.1)	418 (49.9)	774 (52.2)	408 (49.9)	452 (53.9)
Land	996 (69.8)	608 (72.7)	1046 (70.6)	563 (69)	566 (67.5)
Electricity	450 (31.5)	272 (32.5)	480 (32.4)	252 (30.9)	233 (27.8)

1 Data are from reference 17. Plumpy'doz is manufactured by Nutriset. CFC, child feeding counseling; HFI, household food-insecurity score; LAZ, length-for-age *z* score; LSI, living standards index of socioeconomic status; SSC, secondary school certificate; WAZ, weight-for-age *z* score; WLZ, weight-for-length *z* score; WSB++, fortified wheat-soy blend.

2 Determined with the use of a generalized estimating equation linear regression analysis for differences by group (*P-*age < 0.0001).

3 Mean ± SD (all such values)

4 Determined with the use of generalized estimating equation logistic regression analysis for differences by group (*P*-stunting = 0.03).

5 Determined with the use of generalized estimating equation multinomial logistic regression analysis (*P*-cattle ownership = 0.009).

### Breastfeeding

**FIGURE 2:**
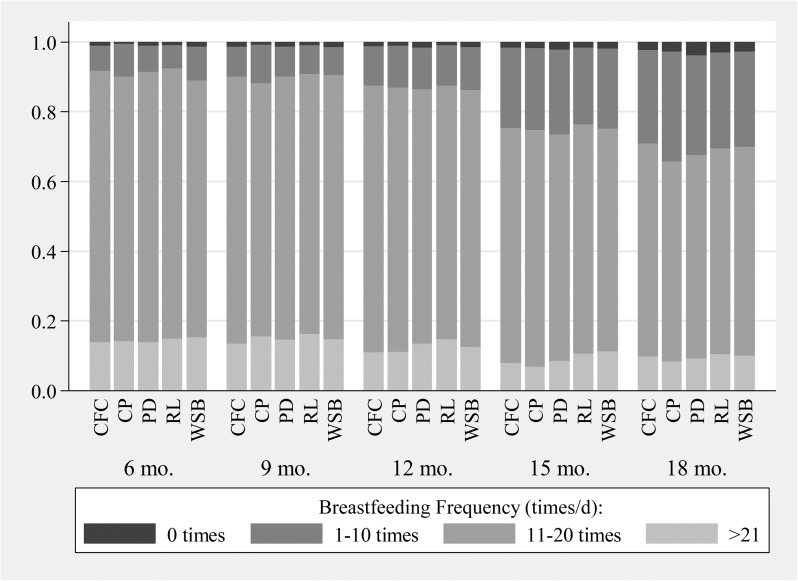
Distribution in breastfeeding-frequency categories by supplementation group and age of participants in a randomized controlled trial of complementary food supplements in rural Bangladesh. Breastfeeding frequency did not differ by supplementation group at any age (*P* > 0.50) according to ordinal logistic regression models with SEs adjusted for design effects. Sample sizes ranged from *n* = 5384 at the 6-mo interview to *n* = 5066 at the 18-mo interview. CFC, child feeding counseling; CP, chickpea complementary food supplement; PD, Plumpy'doz complementary food supplement (Nutriset); RL, rice and lentil complementary food supplement; WSB, fortified wheat-soy blend.

Rates of breastfeeding remained high throughout the study period (98.7% and 97.1% of children were breastfed at 12 and 18 mo of age, respectively), and neither the discontinuation nor frequency of breastfeeding differed by CFS assignment (*P* > 0.50) (**Figure 2**).

### Home diet

**FIGURE 3:**
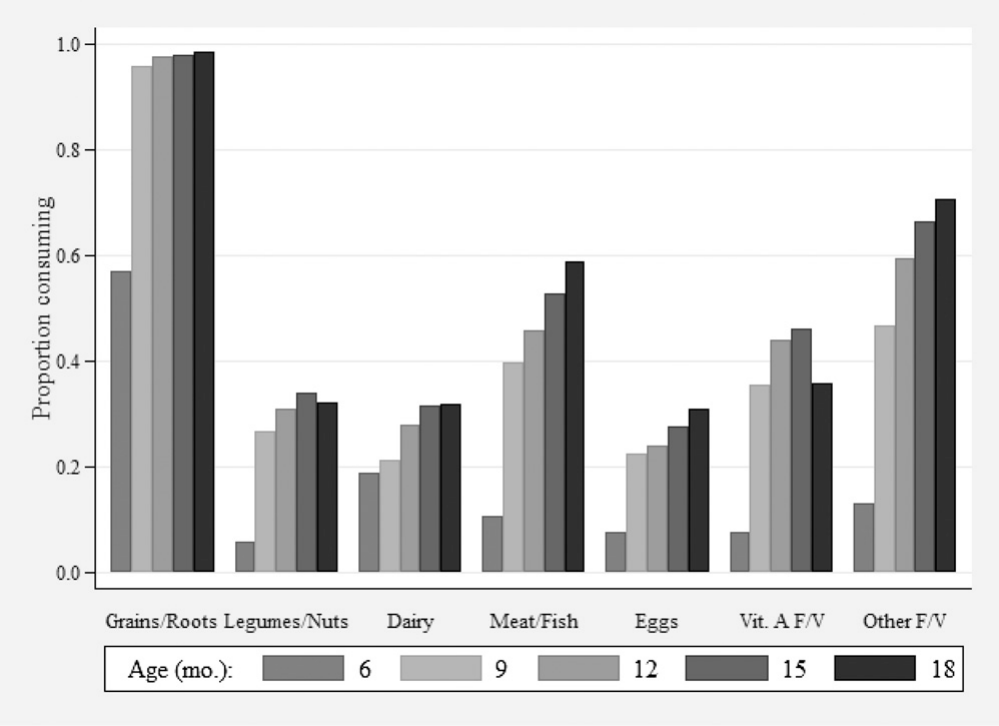
Proportion of children enrolled in a randomized trial of complementary food supplements in Bangladesh who consumed each of 7 food groups, by age at the time of interview. Sample size ranged from 5384 at the 6-mo interview to 5067 at the 18-mo interview. F/V, fruit and vegetables; Vit. A F/V, vitamin A–rich fruit and vegetables.

Grains and starchy roots was the most-frequently consumed food group at all ages followed by meat, non–vitamin A–rich fruit and vegetables, and vitamin A–rich fruit and vegetables, whereas legumes and nuts, dairy, and eggs were consumed least frequently (**Figure 3**). The consumption of all food groups increased with age, most markedly between ages 6 and 9 mo. Individual foods that were consumed within each food group were largely stable over the ages observed (**Table 2**). Khichuri, which is a traditional mixed dish of rice, lentils, and vegetables, was reportedly consumed in the past 24 h by <10% of children at all ages but was a top contributor to consumption of legumes and nuts, vitamin A–rich fruit and vegetables, and other fruit and vegetables. The rate of animal-milk consumption was low at all ages (<25%) and peaked at 15 mo of age with a small decline between 15 and 18 mo of age. Fish was the most frequently consumed type of flesh food (consumed by 45% of children at 18 mo of age) followed by meat and chicken (consumed by 18% of children at 18 mo of age), but liver consumption also contributed to that food group (consumed by 8–10.5% of children at 9–18 mo of age) especially at younger ages when the consumption of meat and chicken was less common. Biscuits (cookies), cake, candy, and sweet fried snacks (e.g., gilapi and khorma), which did not contribute to the DDS calculation, were also frequently consumed at all ages (27.7% and 56.5% of children consumed biscuits at ages 6 and 18 mo, respectively). By age 9 mo, 31.3% of children had achieved the MDD, whereas 42.8%, 51.0%, and 51.4% of children reached the MDD threshold at 12, 15 and 18 mo of age, respectively, across the 5 study arms.

**TABLE 2:** Most frequently consumed foods within each food group by age at interview in children participating in a randomized trial of complementary food supplements in rural Bangladesh^1^

	Food item (%)				
Food group	6 mo	9 mo	12 mo	15 mo	18 mo
Grains and starchy roots	Rice (42.7)	Rice (92.5)	Rice (95.2)	Rice (95.5)	Rice (95.7)
	Suji or payesh (17.9)	Potato (65.5)	Potato (74.6)	Potato (73.5)	Potato (77.1)
	Potato (12.4)	Puffed rice (22.2)	Puffed rice (33.4)	Puffed rice (39.0)	Puffed rice (42.2)
Legumes/nuts	Dal (4.0)	Dal (21.8)	Dal (25.8)	Dal (28.1)	Dal (24.4)
	Khichuri (2.2)	Khichuri (7.1)	Khichuri (6.6)	Khichuri (6.2)	Khichuri (6.7)
	Peanuts (0.0)	Singara (0.3)	Singara (0.8)	Singara (1.6)	Singara (2.6)
Dairy	Milk (8.7)	Milk (13.6)	Milk (19.8)	Milk (24.5)	Milk (23.0)
	Suji or payesh (6.2)	Suji or payesh (7.5)	Suji or payesh (9.0)	Suji or payesh (8.6)	Suji or payesh (10.0)
	Formula (5.2)	Formula (1.9)	Yogurt (2.3)^2^	Yogurt (3.0)	Yogurt (3.7)
Meat and fish	Fish (7.4)	Fish (30.0)	Fish (34.9)	Fish (39.3)	Fish (45.3)
	Liver (3.3)	Liver (8.6)	Meat (11.9)	Meat (16.1)	Meat (18.0)
	Meat (0.9)^3^	Meat (7.1)	Liver (8.8)	Liver (10.5)	Liver (10.1)
Eggs	Egg (7.5)	Egg (22.3)	Egg (23.8)	Egg (27.5)	Egg (30.8)
	Noodles (0.2)^4^	Noodles (0.4)	Noodles (0.3)	Noodles (0.5)	Noodles (0.4)
Vitamin A–rich F/Vs	Leafy vegetables (5.0)	Leafy vegetables (21.8)	Leafy vegetables (28.5)	Leafy vegetables (28.8)	Leafy vegetables (27.3)
	Khichuri (2.2)	Khichuri (7.1)	Mango (9.6)	Mango (15.8)	Khichuri (6.7)
	Papaya (0.4)	Mango (6.3)	Khichuri (6.6)	Khichuri (6.2)	Papaya (2.6)
Other F/Vs	Banana (3.9)	Eggplant (14.0)	Eggplant (16.8)	Eggplant (23.5)	Eggplant (27.6)
	Khichuri (2.2)	Banana (8.2)	Banana (14.2)	Banana (21.2)	Banana (22.7)
	Eggplant (2.0)	Khichuri (7.1)	Parble (12.2)	Parble (18.0)	Parble (10.7)
Other^5^	Biscuits (27.7)	Biscuits (43.8)	Biscuits (48.7)	Biscuits (50.4)	Biscuits (56.5)
	Cake (5.3)	Cake (14.4)	Cake (17.4)	Cake (19.8)	Cake (23.3)
	Candy (3.0)	Candy (4.7)	Gilapi (8.5)	Gilapi (12.2)	Candy (15.0)

1 Values in parentheses are percentages of children who consumed each food in the past 24 h. Sample sizes ranged from *n* = 5384 at 6 mo of age to *n* = 5067 at 18 mo of age. F/V, fruit and vegetable.

2 Yogurt included yogurt and dairy-based sweets.

3 Meat included beef, goat, and chicken.

4 Noodles refers to a stir-fried mixed dish containing noodles, egg, and vegetables.

5 Foods in this group were not counted toward the dietary diversity score. Note that biscuits are cookies and gilapi is one of several types of fried sweet or salty snack foods that were all counted within this food item.

### Supplementation effects

Supplementation with CFSs was associated with an equal or increased odds of reaching the MDD with home foods (**Table 3**). Supplementation with chickpea, Plumpy'doz, and WSB++ was positively associated with achieving an MDD from home foods across the ages studied, whereas consumption of the rice and lentil CFS trended in the same direction but did not reach statistical significance (*P* = 0.13). The absolute difference in the percentage of children who achieved the MDD, averaged across the interview time points, in the chickpea group relative to the CFC-only group was 4.1% (95% CI: 0.7%, 7.5%). The magnitude of effect in the Plumpy'doz and WSB++ groups was similar, whereby the percentage of children who reached the MDD was higher by 4.0% (95% CI: 1.2%, 6.9%) and 4.2% (95% CI: 0.9%, 7.5%) in the Plumpy'doz and WSB++ groups, respectively.

**TABLE 3:** Effect of complementary food supplementation on the prevalence of MDD by interview age in children participating in a randomized controlled supplementation trial in rural Bangladesh (*n* = 5396)^1^

	CFC only	Chickpea	Plumpy'doz	Rice and lentil	WSB++
*n*	1436	839	1490	823	849
MDD, age in mo, %					
9	29.1	35.1	30.6	31.8	32.1
12	39.0	46.0	44.0	43.0	44.0
15	48.3	52.4	52.3	50.2	52.8
18	48.7	49.2	54.8	50.2	53.0
Difference (95% CI)^2^	—	4.1 (0.7, 7.5)**	4.0 (1.2, 6.9)***	2.6 (−0.7, 6.0)	4.2 (0.9, 7.5)**

1 Complementary food supplements were not included in the calculation of the MDD. Plumpy'doz is manufactured by Nutriset. CFC, child feeding counseling; MDD, minimum dietary diversity; WSB++, fortified wheat-soy blend.

2 Predicted marginal differences across all ages, *P* values, and 95% CIs were determined with the use of a mixed-effects logistic regression model with MDD as the dependent variable and age, supplementation group, and age × supplementation group interaction terms as independent variables with random intercepts for the child and sector. ** *P >* 0.01 but ≤ 0.05, ***P ≤ 0.01.

Group-wise differences for individual food groups were observed only at 12 mo of age when children in the chickpea, Plumpy'doz, and WSB++ groups reported higher intakes of legumes, and children in the chickpea and Plumpy'doz groups reported greater consumption of non–vitamin A–rich fruit and vegetables than in the control group (data not shown).

### Predictors of diet quality

In univariate analyses, the mother’s education and higher LSI were associated with increased odds of MDD from home foods at age 18 mo, and a higher HFI (more-severe food insecurity) was associated with decreased odds of MDD at 18 mo of age (**Table 4**). However, in multivariable models, only the mother’s education and LSI were each significantly positively associated with the MDD. Compared with children of mothers with no formal education, the ORs of achieving the MDD were 1.63 95% CI: 1.40, 1.90), 2.51 (95% CI: 1.83, 3.46), and 3.72 (95% CI: 2.72, 5.10) in children of mothers with 1–9 y of education, of mothers who passed the secondary school certificate, and of mothers with ≥11 y of education, respectively. Children in households of high SES based on the LSI had 43% (95% CI: 25%, 63%) greater risk of reaching the MDD than children in low-SES households.

**TABLE 4:** Participant and household characteristics predictive of MDD from home foods at age 18 mo (*n* = 5067)^1^

			OR (95% CI)	
	*n* (%)	MDD, *n* (%)	Unadjusted^2^	Adjusted^3^
Mother's education				
None	1210 (23.9)	467 (38.6)	1.00 (1.00, 1.00)	1.00 (1.00, 1.00)
1–9 y	3281 (64.8)	1717 (52.3)	1.75 (1.52, 2.01)	1.63 (1.40, 1.90)
SSC passed	255 (5)	173 (67.8)	3.36 (2.50, 4.51)	2.51 (1.83, 3.46)
≥11 y	310 (6.1)	239 (77.1)	5.36 (4.01, 7.16)	3.72 (2.72, 5.10)
LSI				
Low	2523 (49.8)	1082 (42.9)	1.00 (1.00, 1.00)	1.00 (1.00, 1.00)
High	2544 (50.2)	1520 (59.7)	1.98 (1.77, 2.21)	1.43 (1.25, 1.63)
HFI				
None	2609 (51.5)	1462 (56)	1.00 (1.00, 1.00)	1.00 (1.00, 1.00)
Mild	1817 (35.9)	877 (48.3)	0.73 (0.65, 0.83)	0.96 (0.84, 1.10)
Severe	637 (12.6)	262 (41.1)	0.55 (0.46, 0.65)	0.87 (0.72, 1.06)
Sex				
M	2526 (49.9)	1307 (51.7)	1.00 (1.00, 1.00)	1.00 (1.00, 1.00)
F	2541 (50.1)	1295 (51)	0.97 (0.87, 1.08)	0.96 (0.86, 1.08)

1 Complementary food supplements were not included in the calculation of the MDD. HFI, household food-insecurity score; LSI, living standards index of socioeconomic status; MDD, minimum dietary diversity (dietary diversity score ≥4); SSC, secondary school certificate.

2 Univariate models are logistic regressions with the MDD as the outcome and indicator variables for levels of one of the child or household characteristics as predictors with SEs adjusted for design effect.

3 Multivariable models are logistic regressions with the MDD as the outcome and indicator variables for all listed child and household characteristics, adjusted for these other maternal and household characteristics: child age, assigned supplementation group, breastfeeding continuation to 18 mo of age (yes or no), number of household members, maternal employment (working outside of the home compared with not working outside of the home), maternal age, household land ownership, and ownership of cattle, goats and sheep, and chickens with SEs adjusted for design effect.

## DISCUSSION

In children 6–18 mo of age who were enrolled in a randomized controlled trial of complementary food supplementation, the assessment of home-food dietary intakes revealed low dietary diversity. Diets in these infants and young children were primarily comprised of rice, potato, and biscuits (cookies), whereas the consumption of animal-source foods and vitamin-rich fruit and vegetables was less common. Daily supplementary feeding with 1 of 4 types of fortified complementary foods, with high adherence, in combination with CFC improved the diversity of the diet provided at home and did not alter the frequency of breastfeeding compared with controls who received no food supplements but whose mothers received CFC.

In the current study, MDD, which was defined as the consumption of ≥4 different food groups, increased with age but remained low and barely exceeded 50% by 18 mo of age. This suggests widespread risk of inadequate nutrient intakes, which is consistent with the results of other recent studies in the country. One survey by the Alive and Thrive project in Bangladesh reported a mean DDS of 2.9, but only 31.1% of children aged 6–24 mo reached the MDD (26), whereas, in the nationally representative Bangladesh Demographic and Health Survey data, 45% of children aged 6–24 mo reached the MDD (27).

CFSs were not observed to be substituted for typical complementary foods offered in the home or to reduce breastfeeding frequency. These results are in line with those of several recent studies that also detected no negative impact of CFSs on the consumption of typical complementary foods (15, 16, 28) or breastmilk intake (14). In our study, none of the supplemental foods appeared to reduce the breastfeeding frequency or to displace home foods, which may have been attributable to the study procedures, such as the high levels of fieldworker involvement at the household level and effective messaging about feeding supplements in addition to usual breastfeeding and home foods, or to other contextual or cultural factors. The small supplement portion size of only ∼25 and ∼50 g/d for the Plumpy'doz, chickpea, and rice and lentil CFSs (for children aged 6–11 and 12–17 mo, respectively) may also have contributed by making it more feasible to feed the supplement without replacing other home foods or breastmilk. Still, the WSB++ was fed as a bulkier porridge, and it also did not appear to replace other foods, although the quantities consumed could have been affected.

The positive effects of supplementation on the percentage of children meeting the MDD from home foods may occur through a number of pathways. Better nutritional status may, itself, improve appetite (29), and increased energy density of the diet may also have benefits for appetite (13, 30, 31). In addition, better growth and a larger body size may, themselves, increase dietary intake. The introduction of CFSs may have affected the mothers’ feeding behaviors because mothers could have increased their active and responsive feeding behaviors on the basis of encouragement and demonstrations from field workers to feed the full supplement portion daily. In addition, perceptions about the variety and types of flavors and foods that are appropriate for young children, which may influence child feeding practices (32), may have been challenged and expanded by the introduction of the supplements. It is not clear why the rice and lentil food did not increase the proportion of children who reached the MDD in line with the effects of the other CFSs because these proposed pathways of effect would not be expected to differ by CFS type. To our knowledge, this is the first study to report improvements in the maternal-reported child diet with supplementation. Further studies are needed to confirm these findings and to investigate potential pathways in greater depth than our data allow.

In this rural Bangladeshi cohort, higher maternal education and higher SES were independently associated with an increased likelihood of children achieving the MDD. Associations between child diet quality and maternal education and household SES have been observed previously in Bangladesh and elsewhere (33, 34) as have relations between child nutritional status and maternal education and household SES (35). These findings are also in line with evidence that maternal education has benefits for child health that are independent of household economic means. Taken together, these results suggest that, pending broader improvements in household socioeconomic characteristics, CFSs may provide critical nutrients to the least-advantaged children who otherwise have high risk of inadequate dietary intakes.

Strengths of this study include the randomized design of the parent trial, which allowed for the isolation of the impact of CFSs on the diet, and the detailed semistructured 24-h dietary questionnaire. In addition, the large sample size and extensive data set of child, maternal, and household characteristics allow for a robust examination of factors that are related to dietary diversity in this setting. Potential limitations include the use of the DDS, which does not contain information about quantities of food or specific nutrients, the lack of quantitative breastfeeding data and the lack of differentiation between full feeds and breastfeeding for pacifying the child, and the use of maternal recall of child food consumption rather than the use of direct observation. In general, an imprecise diet assessment would be expected to have attenuated the observed associations, and broad groupings of breastfeeding frequency and food intakes may have masked more subtle effects of the CFSs on the children’s diets. It is possible that maternal recall and estimation of the child's diet were differentially affected by sociodemographic factors that were also associated with the diet quality such as the mother’s education and the household’s food-security status, which could have amplified the observed associations between those characteristics and dietary intakes. Previous research has shown interviewer-administered dietary questionnaires to be reliable in a variety of populations (36), reducing the likelihood that this aspect was a source of bias. It is possible that, in the study arms that received CFSs, mothers were more likely to overreport the child’s dietary intake because of the messaging from study personnel. The CFC that was received by mothers in all groups contained messages regarding appropriate complementary feeding practices, which may have reduced the likelihood that the study messaging or interactions with study personnel had a differential impact on the mother’s report of child diet. In addition, the study personnel who were responsible for the distribution of CFSs and who conducted the CFC were distinct from the cadre of interviewers, which further reduced the likelihood of a differential reporting bias in the supplemented compared with unsupplemented groups.

In conclusion, the low dietary diversity observed in children in this study further supports the contribution of inadequate diets in the fragile complementary feeding period to the prevalent stunting that is observed in this setting and underscores the potential for CFSs to help ameliorate stunting, as was shown in the parent trial, by supplying children with the nutrients that they lack for healthy growth. Feeding practices around CFSs may differ markedly by cultural and household context, but in this large sample in a rural South Asian setting, supplemental foods of multiple types did not appear to be substituted for other complementary foods or to displace breastfeeding, which has been posed frequently as a potential concern, but, on the contrary, may have led to some improvements in the diversity of home foods being provided to children receiving these supplements. In combination, these findings support growing evidence that CFSs may be necessary and appropriate in the rural South Asian context.

## Footnotes

7 Abbreviations used: CFC, child feeding counseling; CFS, complementary food supplement; DDS, dietary diversity score; HFI, household food insecurity index; LAZ, length-for-age *z* score; LSI, living standards index; MDD, minimum dietary diversity; SES, socioeconomic status; WSB++, fortified wheat-soy blend.
